# Performance of standardized cancer patient pathways in Sweden visualized using observational data and a state-transition model

**DOI:** 10.1038/s41598-023-46757-x

**Published:** 2023-11-09

**Authors:** Sixten Borg, Ann-Sofi Hörstedt, Tobias Carlsson, Mef Nilbert, Anna-Maria Larsson, Björn Ohlsson

**Affiliations:** 1 Regional Cancer Centre South, RCC Syd, Scheelevägen 8, 223 81 Lund, Sweden; 2 Health Economics Unit, Department of Clinical Sciences in Malmö, Lund University, Lund, Sweden; 3 Division of Oncology, Department of Clinical Sciences, Skåne University Hospital, Lund University, Lund, Sweden; 4 Department of Surgery, Blekinge Hospital, Karlshamn, Sweden

**Keywords:** Cancer, Diagnosis, Health policy, Health services

## Abstract

Standardized Cancer Patient Pathways (CPPs) were introduced in Swedish healthcare starting in 2015 to improve diagnostics for patients with symptoms of cancer, patient satisfaction and equity of care between healthcare providers. An inclusion target and a time target were set. Our primary aim was to visualize the patient population going through CPPs, in terms of investigation time and indications of the various outcomes including cancer diagnoses. Our secondary aims were to examine if targets were met, and to examine frequencies of undetected cancer. We collected data from 19,204 patients starting in a CPP, and 7895 patients diagnosed with cancer in 2018 in a region of Sweden. A state transition model was developed and used as analytical framework, and patients were mapped over time in the states of the model. Visualization of the patient-flow through the model illustrates speed of investigation, time to treatment, frequencies of detected and undetected cancer. Twelve CPPs out of 28 met the inclusion target, five met the time target. After suspicion of cancer rejected, 0.8% of patients were diagnosed with the primarily suspected cancer, 1.0% with another cancer. In patients not meeting the criteria for well-founded suspicion less than 3% were later diagnosed with cancer. The visualization of the patient flow into and through standardized cancer patient pathways illustrates investigation time, events occurring and outcomes. The use of standardized cancer patient pathways detects cancer efficiently.

## Introduction

In 2015, the Swedish government started to implement standardized cancer patient pathways (CPPs) to reduce time to cancer treatment, improve patient satisfaction and increase equity in cancer care^1^. Previously, CPPs had been implemented elsewhere in Europe. The Swedish implementation was based on the Danish model, though adapted to our setting^2^, and between 2015 and 2018, 31 CPPs were implemented^3^.

Each CPP defines symptoms and signs required for well-founded suspicion of cancer, principles for referral to specialized care and requested pre-diagnostic investigations. Some CPPs have a filter function, meaning that well-founded suspicion must be confirmed by a specialist. Most CPPs have a lead time from well-founded suspicion to start of treatment. Data on dates and outcomes of CPPs (e. g. start of treatment, suspicion of cancer rejected) are recorded and monitored on a national basis.

Two national CPP targets were defined^4^: the inclusion target dictates that at least 70% of patients diagnosed with cancer should be investigated within the CPP covering that diagnosis. The lead time target dictates that at least 80% of patients included should start on treatment within a diagnosis and treatment modality specific lead time. Our alternative phrasing for the purpose of studying investigation time overall, was that for at least 80% of the patients, the investigation though a CPP should end within the defined lead time^3^, i. e. be it treatment start, or suspicion rejected.

The targets indicate if CPPs are employed and whether the investigation is carried out within recommended times frames. It also appears reasonable to examine if cancer is discovered efficiently and whether CPP inclusion criteria are defined well according to medical need, healthcare resource consumption and patient experiences.

There are to date no systematic and comprehensive descriptions of the events and outcomes of CPPs, across different CPPs, using individual patient data, in Sweden or in the Nordic countries. Some aspects have been described, based on aggregate data from annual reports, and interviews of healthcare staff^2, 5–7^, or in studies on specific CPPs^8, 9^. For instance, Schmidt et al. found reduced waiting times early during CPP implementation^5^. We used individual data on patients going through CPPs, deterministically linked to cancer diagnoses from the National Cancer Register (cancer register). Hereby we hope to provide a systematic description of the CPPs along with insights on target fulfilment and whether cancer is discovered efficiently.

Our primary aim was to systematically illustrate the event history before, during and after a CPP. We developed a model-based graphic presentation that describes the patients' times and events as they pass through the CPP, and the outcome at its termination. As secondary aims, we evaluated the inclusion and lead time targets, and we also investigated how often it occurred that a patient was diagnosed with cancer despite the suspicion of cancer being previously rejected, or that a patient was diagnosed with cancer after failing to meet the inclusion criteria of a CPP.

The study has been approved by the Regional Ethics Review Board in Lund (Regionala Etikprövningsnämnden EPN Lund, Avdelning 1), Lund, Sweden (Reference number 2016/524).

## Material and method

### Setting and inclusion criteria

We wished to analyse all CPPs whose inclusion criteria and primarily suspected diagnosis were distinguishable in the cancer register, by including all adult patients in the Swedish healthcare region of Skåne who either underwent a CPP, or were diagnosed with cancer, or both, in 2018.

By 2018, 31 CPPs had been implemented. However, Acute Myeloid Leukaemia and Acute Lymphocytic Leukaemia were combined into a single CPP, as were Malignant Lymphomas and Chronic Lymphatic Leukaemia. Furthermore, cases that match the Ovarian cancer CPP inclusion criteria cannot be identified in the cancer register alone, it also requires quality register data^10, 11^. Hereby, the remaining 28 CPPs were selected for analysis in the study. To clarify the distinction between three of the CPPs, the Abdominal and gynaecological sarcoma CPP is used to manage intra-abdominal sarcoma including gastrointestinal stromal tumour, abdominal wall sarcoma and gynaecological sarcoma. The Bone and soft tissue sarcoma CPP includes other sarcoma, i. e. soft tissue sarcoma elsewhere and bone sarcoma, except soft tissue sarcoma in the head and neck region are managed using the Head and neck cancer CPP.

Most CPPs have a primarily suspected diagnosis (PSD), e. g. lung cancer in the Lung cancer CPP, though the CPPs Nonspecific symptoms possibly indicative of cancer, and Cancer with unknown primary tumour (CUP) have no PSD, resulting in a total of 26 PSDs in the study.

### Data collection

In Skåne, individual data are recorded for anyone investigated through a CPP. We obtained data from all adults (≥ 18 years) living in the region who started in a CPP in 2018, resulting in 17 926 individual patients and 19 204 CPP referrals.

In Sweden, the cancer register was founded in 1958 as a national population-based cancer register with the purpose of describing cancer morbidity and change over time and providing a basis for clinical and epidemiological research^12^. We collected data on cancer diagnoses from all adults living in Skåne at the time of diagnosis, between 1 December 2017 and 31 December 2018. The interval covered one month ahead of the date of diagnosis. This resulted in 16 256 diagnoses in 14 461 individual patients, including one or more tumours per individual.

### Detection of cancer cases

We used diagnosis codes, histology and morphology codes to identify and group cancer diagnoses^10, 11^, separating Pancreatic cancer (diagnose code C25.0-3 or 8-9) from Hepatobiliar cancer (C170, C239, C24*). We used the code C80.9, Malignant neoplasm, primary site unspecified, to detect CUP. Benign tumours were placed in the category *Other*.

For a case to be registered in the cancer register, it requires a clinical diagnosis to be confirmed by a pathological diagnosis.

### State-transition model

**Figure 1:**
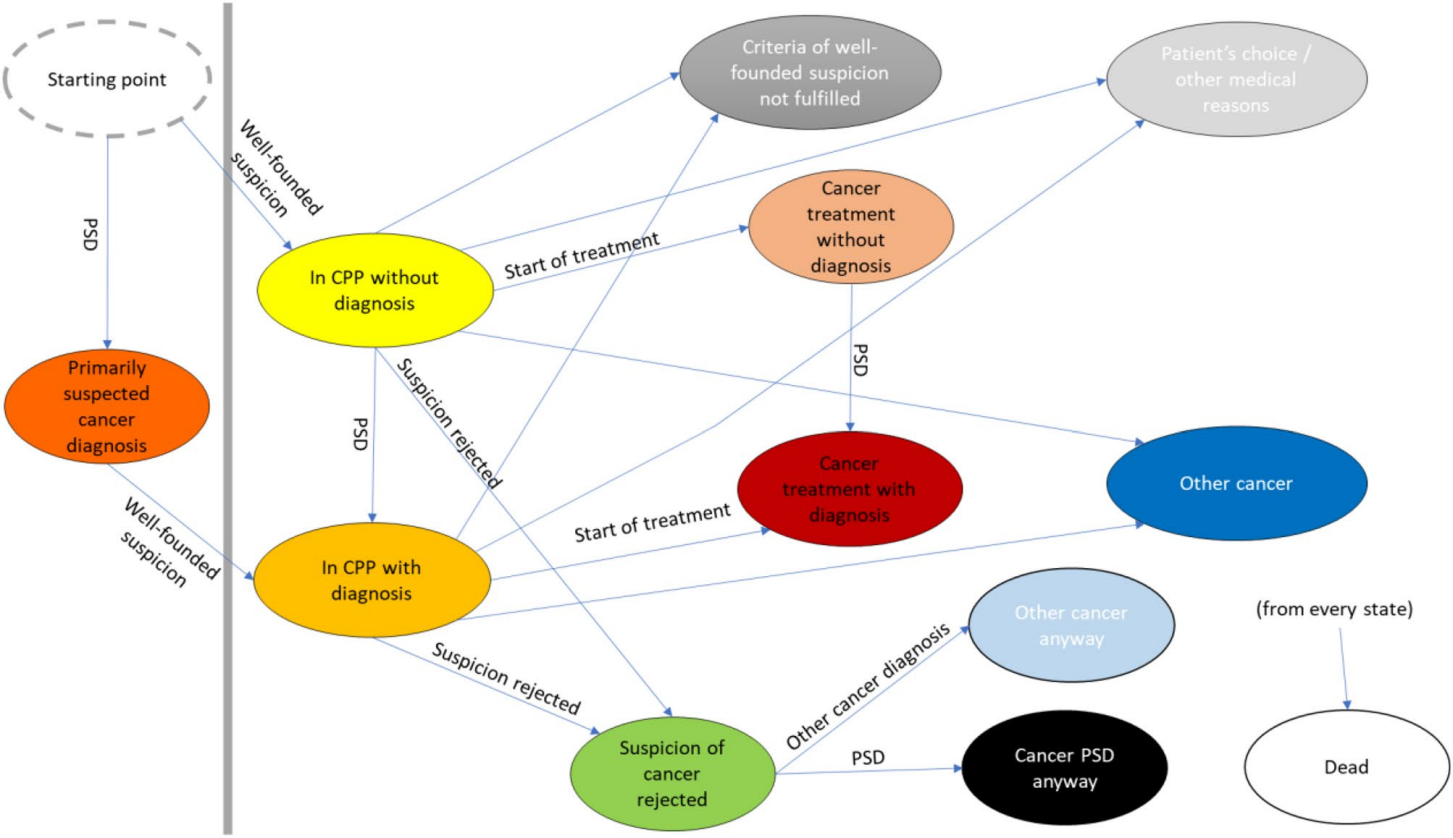
The state-transition model structure used as analytic framework. States (ellipses) and transitions (arrows). *CPP* standardized cancer patient pathway. States left of the grey bar illustrates registration of Primarily Suspected Diagnosis (PSD) before well-founded suspicion (WFS); states to the right are used to illustrate patient flow following WFS.

We defined a state-transition model as a framework for analysing the event histories of patients going through a CPP (supplementary material)^13^. This model design is useful for studying how patients move through different states, at different rates and along different pathways, and for studying and illustrating time spent in the states. At each point in time, a patient was mapped into a state, or as making a transition between two states (Fig. 1). The states and transitions were defined by diagnoses in the cancer register, the date of well-founded suspicion and the CPP stop date (indicating start of treatment, suspicion of cancer rejected, other cancer, criteria for well-founded suspicion not met, patient choice, and other medical reasons). Finally, death (recorded in the cancer register) is a state.

Some states are split in two, depending on whether the patient has the PSD or not, namely *In CPP* without/with diagnosis, and *Cancer treatment* without/with diagnosis. We found that many patients were diagnosed before the date of well-founded suspicion. Including only diagnoses after well-founded suspicion would have inflated the number of CPPs not leading to diagnosis. Our separation of some states depending on diagnosis, i. e. without/with diagnosis, accommodated diagnoses both before and after well-founded suspicion (Fig. 1). Additionally, cancer diagnosed after rejecting the suspicion of cancer are separated into states for PSD and other cancers. In some states, the transition probabilities needed to be dependent on time since entry, which was modelled as in a previous study^14^. Finally, since both a clinical and pathological diagnosis is required for registration in the cancer register, patients with a clinical diagnosis but without pathological confirmation will appear to lack a diagnosis in our analysis.

Using this framework, we illustrated the flow of patients starting with well-grounded suspicion, for all 28 CPPs pooled and for each CPP separately. As an overview, we described the patient flow in the pooled CPPs by stratifying by age (< 70, ≥ 70), sex and CPPs with a filter function versus CPPs without. Inevitably this stratification is influenced by age-related cancer incidence, selection of male/female cancer, and the effect of using a filter function.

### Inclusion target

We examined if the CPPs met the inclusion target by determining if at least 70% of patients diagnosed with the corresponding PSD had been investigated through the CPP. This is feasible in a retrospective study such as ours, however the official near real-time monitoring of the inclusion target uses a different methodology (see “Discussion”)^4, 15^.

### Lead time target

Most of the CPPs have specified lead times from well-founded suspicion to start of treatment, which are the basis for the lead time target. The lead times sometimes differ by treatment modality; though we lack data on the type of treatment and make a conservative analysis using each CPP's longest lead time.

Among the CPPs that did not meet the lead time target, we selected the ten most used to study excess investigation time. Hereby investigation time was partitioned into *within lead time*, and *excess time*.

### Observed cancer cases

For each CPP, we tabulated patients diagnosed with cancer, the PSD and other, thus detected within the CPP. To study undetected cancer, we tabulated patients with suspicion of cancer rejected and those subsequently diagnosed with cancer, PSD and other. Similarly, we also tabulated patients not meeting the criteria for well-founded suspicion and those subsequently diagnosed with cancer.

### Statistical methods

Descriptive statistics on number of patients, number of CPPs and number of diagnoses, and outcomes classified according to the state-transition model framework are presented in tables. In addition, we present lead times, as observed and hypothetically if within lead time target. In the analysis of excess investigation time, by CPP we linearly rescaled the set of observed times, such that they met the lead time target, and within lead time mean investigation time was computed.

Each CPP was analysed individually, and we also pooled all CPPs in an analysis. In the latter, we also made a descriptive stratification by sex, age, and presence of filter function.

We used the R software for modelling, analyses and illustration of the results^16^.

### Ethics approval and consent to participate

The study has been approved by the Regional Ethics Review Board in Lund (Regionala Etikprövningsnämnden EPN Lund, Avdelning 1), Lund, Sweden (Reference number 2016/524). The ethics review board waived the need to obtain informed consent from subjects or their legal guardians. The study was retrospective, and no experiments were carried out on any humans. The methods applied in the study were carried out in accordance with relevant guidelines and regulations.

## Results

In 2018, most patients, 16 770 (94%) were included in a single CPP, but 1 156 (6%) went through more than one, either different CPPs or the same one more than once. Some of them, 483 (3%), were included in two or more CPPs ongoing simultaneously (further details in Supplementary Table S1). This study includes the 16 596 patients included in precisely one of the 28 CPPs in 2018 (Table 1).

**Table 1:** The number of patients going through each CPP, and following WFS having any cancer diagnosis, PSD, suspicion of cancer rejected; of which later diagnosed (PSD, other cancer), and patients not meeting the inclusion criteria; of which later diagnosed with cancer (% of total number of patients unless otherwise stated).

CPP	Total number of patients^†^	Diagnosis on or after WFS^§^; n (%)	Of which PSD; n (%)	Suspicion rejected^§^; n (%)	Of which later diagnosed, PSD; n (%)*	Other cancer; n (%)*	Patients not meeting criteria for WFS; n (%)	Of which later diagnosed with cancer**; n (%)
Colorectal cancer	3 278	625 (19)	507 (16)	1889 (58)	1 (0.1)	32 (1.7)	38 (1)	1 (3)
Urothelial cancer	2 550	316 (12)	277 (11)	1 858 (73)	6 (0.3)	27 (8.5)	5 (0)	0 (0)
Breast cancer	2 512	1014 (40)	998 (40)	1 308 (52)	3 (0.2)	6 (0.5)	29 (1)	0 (0)
Prostate caner	2 155	551 (26)	524 (24)	1 140 (53)	16 (1.4)	27 (2.4)	13 (1)	1 (8)
Melanoma	1 668	618 (37)	511 (31)	481 (29)	1 (0.2)	13 (2.7)	121 (7)	4 (3)
Lung cancer	944	156 (17)	132 (14)	267 (28)	3 (1.1)	5 (1.9)	12 (1)	0 (0)
Head and neck cancer	678	158 (23)	109 (16)	364 (54)	0 (0.0)	7 (1.9)	2 (0)	0 (0)
Uterine cancer	347	85 (25)	67 (19)	148 (43)	2 (1.4)	1 (0.7)	11 (3)	2 (18)
Nonspecific symptoms possibly indicative of cancer	289	27 (9)	--- (---)	222 (77)	--- (---)	5 (2.3)	8 (3)	0 (0)
Pancreatic cancer	249	91 (37)	64 (26)	37 (15)	0 (0.0)	2 (5.4)	7 (3)	0 (0)
Kidney cancer	231	35 (15)	28 (12)	55 (24)	1 (1.8)	2 (3.6)	0 (0)	… (…)
Lymphoma	221	65 (29)	30 (14)	34 (15)	0 (0.0)	1 (2.9)	18 (8)	0 (0)
Cancer with unknown primary tumour	220	55 (25)	--- (---)	72 (33)	--- (---)	5 (6.9)	5 (2)	0 (0)
Oesophageal and stomach cancer	191	122 (64)	110 (58)	30 (16)	0 (0.0)	0 (0.0)	4 (2)	1 (25)
Brain tumour	145	66 (46)	55 (38)	25 (17)	0 (0.0)	1 (4.0)	13 (9)	0 (0)
Bone and soft tissue sarcoma	124	7 (6)	5 (4)	98 (79)	0 (0.0)	0 (0.0)	1 (1)	0 (0)
Testicular cancer	118	42 (36)	41 (35)	69 (58)	0 (0.0)	0 (0.0)	0 (0)	… (…)
Thyroid cancer	114	24 (21)	22 (19)	2 (2)	0 (0.0)	0 (0.0)	1 (1)	0 (0)
Cervix cancer	95	23 (24)	19 (20)	18 (19)	0 (0.0)	0 (0.0)	1 (1)	0 (0)
Liver cancer	92	21 (23)	18 (20)	15 (16)	0 (0.0)	1 (6.7)	3 (3)	0 (0)
Myeloma	88	42 (48)	38 (43)	16 (18)	0 (0.0)	1 (6.2)	12 (14)	0 (0)
Hepatobiliary cancer	80	10 (13)	8 (10)	25 (31)	1 (4.0)	0 (0.0)	2 (3)	0 (0)
Anal cancer	55	9 (16)	8 (15)	21 (38)	0 (0.0)	0 (0.0)	4 (7)	0 (0)
Penile cancer	48	9 (19)	9 (19)	26 (54)	0 (0.0)	0 (0.0)	0 (0)	… (…)
Vulvar cancer	42	6 (14)	5 (12)	10 (24)	0 (0.0)	0 (0.0)	3 (7)	0 (0)
Acute leukaemia	34	25 (74)	23 (68)	2 (6)	0 (0.0)	1 (50.0)	0 (0)	… (…)
Abdominal and gynaecological sarcoma	19	7 (37)	5 (26)	1 (5)	0 (0.0)	0 (0.0)	0 (0)	… (…)
Neuroendocrine tumours	9	3 (33)	2 (22)	1 (11)	0 (0.0)	0 (0.0)	0 (0)	… (…)

*CPP* standardized cancer patient pathway, *WFS* well-founded suspicion, *PSD* primarily suspected cancer.

---  the CPP has no PSD. …  not applicable/not available.

†In addition to patients shown in columns to the right, the total comprises patients diagnosed before WFS, patients starting treatment without diagnosis, patients excluded due to patient's choice or other medical reasons, and patients still under investigation at the end of the time frame.

^§^Within 6 months from WFS.

*Proportion among those with cancer suspicion rejected.

**None of which had the PSD.

The remainder of this section is organized as follows; "Patient flow" pertains to our primary aim and presents visualization of patient flow. The following sections pertain to our secondary aims, "Inclusion target" and "Lead time target" examine the inclusion and lead time targets, and "Observed cancer cases" presents detected and undetected cancer.

### Patient flow

**Figure 2:**
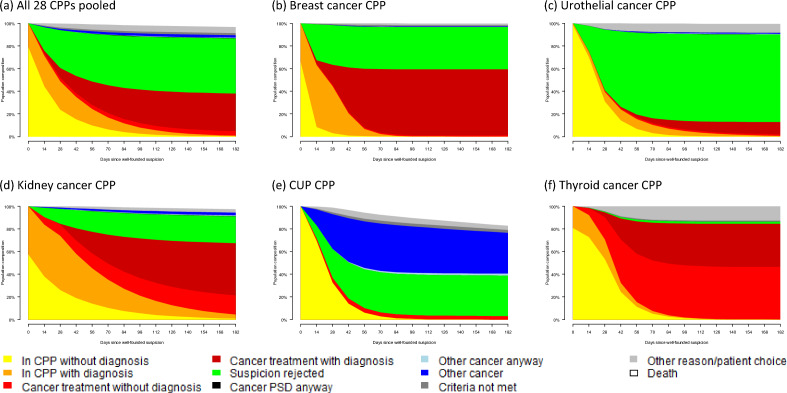
Estimated patient flow following well-founded suspicion (WSF), in all CPPs pooled (**a**), and in five selected CPPs (**b–f**). *CPP* standardized cancer patient pathway, *CUP* cancer with unknown primary tumour.

The patient flow in all 28 CPPs pooled, and the flow in five selected CPPs are presented in Fig. 2. Supplementary figure S1 presents all 28 CPPs individually. In these figures, the coloured areas describe at each time point the proportion of patients visiting each state, the colours corresponding to Fig. 1 (see also "State-transition model"). At day zero, about 80% of the pooled patients start in a CPP without diagnosis, the remainder in CPP with diagnosis (Fig. 2a). After 50 days, the suspicion of cancer has been rejected in about 40%, cancer treatment has begun in 20% and 30% are still under investigation with or without diagnosis. The remainder are spread across the states of Other cancer, Other medical reasons, Patient's choice and Death. Among those with suspicion rejected, 1.8% are subsequently diagnosed with cancer, of which 0.8% with the PSD. This proportion varies between 0 and 4% in the individual CPPs (Table 1). They are illustrated by a black stripe, peaking at 0.24% simultaneously about 100–140 days after well-founded suspicion (Fig. 2a).

We also present five individual CPPs in Fig. 2. In the Breast Cancer CPP, patients are diagnosed quickly, after mean 7 days (going from the yellow to the orange area; Fig. 2b). A large proportion get cancer treatment (light and dark red), 31% after 50 days, and 59% after 100 days. The dark red area represents those starting treatment after diagnosis (98% of treated). The average time to treatment is 43 days. A considerable proportion (35%) start the CPP diagnosed already (orange colour). In this CPP, mammography is a filter function, a suspicious finding calls for clinical examination and decision on well-grounded suspicion.

In the Urothelial Cancer CPP (Fig. 2c), the suspicion of cancer is often rejected (green area), a proportion growing to 77% over time. This may suggest a generous inclusion of patients into the CPP. Only 2% start the CPP with a diagnosis before well-founded suspicion. This CPP does not have a filter function. The investigation is long, average time to diagnosis is 29 days. Among treated, 87% start their treatment after diagnosis. A generous inclusion of patients and long investigation time might indicate high resource use (see discussion).

Investigation through the Kidney Cancer CPP appears slower, half of the patients have either been given a diagnosis, started treatment or had their suspicion rejected by 65 days (Fig. 2d). At CPP start, 42% have a diagnosis already. There is a filter function, primarily a computer tomography scan. Of those who start treatment, 25% do not have a diagnosis (light red area), and of these, 9% are diagnosed later.

The CUP CPP has a big blue area representing patients whose CPP ends with other cancer (Fig. 2d). This CPP lacks a PSD so some states cannot be visited, for instance *In CPP with diagnosis* and *Cancer treatment with diagnosis*, and it appears reasonable that any cancer discovered be coded as other cancer. The observed cancer diagnoses in this CPP are presented in detail in "Observed cancer cases".

Finally, we looked at the Thyroid CPP, which has a distinctly different diagram (Fig. 2f). The majority of patients in the CPP reaches cancer treatment (84%), and only a fraction has their suspicion of cancer rejected (1.8%). There is a filter function comprising ultrasound examination and fine needle aspiration biopsy. As many as 63% of those who starts on treatment do so without diagnosis. In ambiguous cases, surgery is carried out which is coded as treatment in the CPP data, and a diagnosis if any might come later.

In the pooled CPPs, we made a descriptive stratification by sex, age, and filter function (Supplementary Fig. S3, Supplementary Table S2). After 28 days, 54% of males and 45% of females are still being investigated, and 24% of males have the PSD and 9% started treatment (27% and 15% in females). Rejection of cancer suspicion is less frequent in men by 38 days (32 vs 35%) but more frequent after 98 days (50% vs 45%). Fewer patients < 70 years were diagnosed with the PSD after 28 days, 24% compared to 30% in older patients, and more had suspicion rejected, 36% versus 26%. Finally, after 28 days, 43% of patients in CPPs with filter function are still investigated compared to 56% in CPPs without filter function, and 32% has been diagnosed with PSD and 25% started treatment compared to 22% and 4% in CPPs without filter function. The suspicion of cancer has been rejected in 27% and 35% respectively.

### Inclusion target

Out of the 26 CPPs with a PSD, twelve reached the inclusion target that at least 70% of patients diagnosed were investigated through a CPP (Table 2). The average proportion was 74% (range 8–92%).

**Table 2:** Number of diagnoses, diagnosed patients, and patients who started in the corresponding standardized cancer patient pathway (CPP).

Diagnosis	Number of diagnoses	Number of patients	Patients starting in the corresponding CPP; n (%)
Breast cancer	1513	1211	1111 (**92%)**
Melanoma	1379	1291	953 (**74%)**
Prostate cancer	1344	1344	665 (49%)
Colorectal cancer	882	852	683 (**80%)**
Lung cancer	740	733	525 (**72%)**
Urothelial cancer	542	526	320 (61%)
Lymphoma	354	348	207 (59%)
Head and neck cancer	227	222	167 (**75%)**
Oesophageal and stomach cancer	193	193	174 (**90%)**
Kidney cancer	188	179	135 (**75%)**
Pancreatic cancer	169	169	116 (69%)
Uterine cancer	164	164	139 (**85%)**
Thyroid cancer	124	122	55 (45%)
Myeloma	109	109	63 (58%)
Brain tumours	90	90	70 (**78%)**
Hepatobiliary cancer	78	78	25 (32%)
Cervix cancer	74	73	54 (**74%)**
Acute leukaemia	72	72	24 (33%)
Abdominal and gynaecological sarcoma	67	67	10 (15%)
Liver cancer	61	61	40 (66%)
Neuroendocrine tumours	51	51	4 (8%)
Testicular cancer	50	49	43 (**88%)**
Anal cancer	35	35	30 (**86%)**
Penile cancer	34	34	13 (38%)
Bone and soft tissue sarcoma	34	34	9 (26%)
Vulvar cancer	19	19	11 (58%)

The CPPs meeting the ≥ 70% inclusion target are indicated with bold text.

### Lead time target

The proportion of patients reaching cancer treatment within the lead time is presented by CPP in Table 3. Among the 27 CPPs that have specified lead times, five met the target of at least 80% of the patients. The average across CPPs was 47%.

**Table 3:** Standardized cancer patient pathways with lead times and proportion of patients starting cancer treatment within the lead time.

Standardized cancer patient pathways	Lead times (days)*	Proportion^§^ (%)
Colorectal cancer	25–39	36
Urothelial cancer	22–43	22
Breast cancer	28	47
Prostate caner	47–68	42
Melanoma	33–57	**95**
Lung cancer	30–44	30
Head and neck cancer	18–38	74
Uterine cancer	25–39	64
Pancreatic cancer	22–36	44
Kidney cancer	27–41	24
Lymphoma	18–26	55
Cancer with unknown primary tumour	24–35	**85**
Oesophageal and stomach cancer	24–38	36
Brain tumour	37–97	**97**
Bone and soft tissue sarcoma	28–39	**81**
Testicular cancer	31–38	51
Thyroid cancer	31	36
Cervix cancer	11–25	19
Liver cancer	22–55	41
Myeloma	15–20	73
Hepatobiliar cancer	22–36	49
Anal cancer	32–46	25
Penile cancer	17–31	44
Vulvar cancer	20–34	60
Acute leukaemia	9	**97**
Abdominal and gynaecological sarcoma	28–39	25
Neuroendocrine tumours	35–56	50

*Lead time may depend on treatment modality. We used the longest lead time for each CPP (see “Method”).

^§^The lead time target is a proportion at least 80% (indicated with bold text).

**Figure 3:**
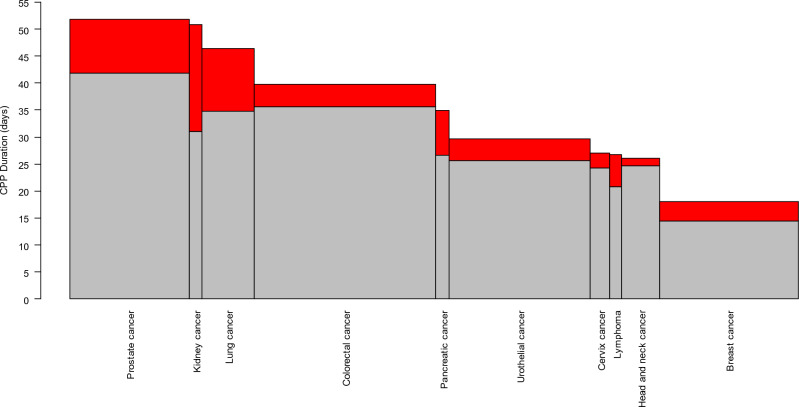
Mean investigation time (days; grey + red), and hypothetical investigation time had the lead time target been met (grey), in the ten most common CPPs that did not meet the lead time target. CPP = standardized cancer patient pathway. The bar widths indicate the number of patients going through the CPPs.

Figure 3 illustrates the ten most used CPPs that did not meet the lead time target. Each CPP has a bar whose width indicates the number of patients going through the CPP, and the total height shows the average investigation time. The red portion indicates the excess time due to not meeting the lead time target. The total excess time in these ten CPPs account for 90% of the total excess time in all CPPs. See discussion.

### Observed cancer cases

The studied CPPs are summarized in Table 1, and further details are given in Supplementary Table S3. The proportion of patients diagnosed with cancer after the start of a CPP varies between 6 and 74%, specifically the PSD varied between 4 and 68%.

We were particularly interested in patients whose suspicion of cancer were rejected, but who were subsequently diagnosed with cancer. The PSD was given in this way to 4% of the patients in the Hepatobiliar cancer CPP, in 1.8% in the Kidney Cancer CPP and on average 0.8% across all CPPs (Table 1). The proportion of patients given other diagnoses than the CPP was 8.5% in the Urothelial cancer CPP and usually fewer in other CPPs, with the exception of 50% in the Acute leukaemia CPP (one of just two patients). The average proportion across all CPPs was 1.0%.

The highest proportions of patients not meeting criteria for well-founded suspicion was 14% in the Myeloma CPP and 9% in the Brain tumour CPP. Very few patients not meeting the criteria were later diagnosed with cancer, in total 9 patients across all CPPs, though due to small numbers the proportion in the individual CPPs varied between 0 and 25%. None of the diagnoses however were the PSD.

A total of 55 patients going through the CUP CPP were diagnosed with cancer, namely Acute Myeloid Leukaemia (1), Breast cancer (5), Lung cancer (5), Lymphoma/Chronic Lymphocytic Leukaemia (2), Oesophageal and stomach cancer (1), Myeloma (1), Neuroendocrine tumours (5), Kidney cancer (1), pancreatic cancer (1), prostate cancer (1), colorectal cancer (2). Another 15 patients received the diagnosis C80.9 without any signs of identified tumours such as histological codes, and 17 received some other diagnosis. The ICD code C80.9 represents true CUP, thus seen in 6.8% of patients starting in the CUP CPP.

## Discussion

Our primary aim with this study was to develop an overview picture of the flow of patients into and through CPPs. Using a state-transition model as a framework, we developed a graphic illustration in the form of a diagram. The diagram shows how fast the patients are investigated, how many patients start the CPP already diagnosed, and the various outcomes of investigation through a CPP. The proportions of patients starting cancer treatment, and having the suspicion of cancer rejected, illustrate important overall outcomes. These are however also dependent on the CPP's inclusion criteria, the presence of a filter function, and how difficult it is to discover the cancer in question. The inclusion criteria might be influenced by a trade-off between the urgency of early discovery of the cancer and the need for healthcare resources. The overview picture illustrates the event history of the patient population, and its outcomes, dynamically over time.

The overview differed between men and women, between younger (< 70 years) and older, and between CPPs with or without filter function. Investigation seems to take longer for men and fewer men than women are diagnosed with cancer. Younger patients have less cancer, and the time to investigate is shorter and more often leads to diagnosis and treatment in CPPs with filter function. These stratifications inevitably lead to selection of male and female cancer, age-dependent cancer incidence and the cancer forms whose CPPs have a filter function. This is a likely cause for our results, however it still appears reasonable to describe these findings. The filter function, for instance, means that specific clinical information indicative of cancer is available already when the CPP starts, and consequently this affects our measurement of investigation time, and subsequent outcomes.

We saw two common CPPs with quite different overview pictures, the Breast cancer CPP with a high proportion starting treatment, and the Urothelial cancer CPP with a high proportion suspicion rejected. From a healthcare resource point of view, this might raise questions. The former CPP has a filter function contrary to the latter. The latter CPP includes patients on basis of symptoms either harmless or indicative of cancer, which is judged motivates investigation. We will return to this discussion in connection to the lead time target.

There is a clearly visible share of patients in both the Kidney cancer and Thyroid cancer CPPs who start cancer treatment without a cancer diagnosis. In some cases, treatment is started on basis of diagnostic imaging. With no pathological verification, the diagnosis is not registered in the cancer register, and our approach identifies this situation as treatment without a diagnosis.

While we claim the diagrams provide a good overview of the CPPs, they also have limitations. The diagrams depend on the design of our state-transition model and some aspects are simply not observable. For instance, in the Urothelial cancer CPP there is an orange stripe of patients diagnosed but not yet started on cancer treatment. We cannot tell from the diagram if these are many patients briefly, or a few patients during a longer time (which we see in the background data is the case). The additive nature of the diagram, where each area is stacked on top of the previous one may make it difficult to gauge their vertical sizes. Though having each in a separate diagram, or e. g. as coloured lines drawn on top of each other (in a non-additive fashion) may give rise to other difficulties in conveying the overview picture. Another potential limitation worth mentioning is our chosen model design. Though from discussion with clinicians, we have perceived that the state-transition model structure (Fig. 1) was acceptable. The specific choice of a Markov model should have very limited impact since we mainly use the model to map patients and visualize patient flow, which we would expect be very similar had we used some other underlying modelling approach. In any case, our model approach allows us to handle censored data and competing events such that we can determine the patient flow through the model, in a range of different CPPs, each managed individually, but using a common model structure.

In general, there were very few cases of cancer suspicion rejected, followed by a PSD. Among those few starting in a CPP but cancelled for not meeting the inclusion criteria, and later diagnosed with cancer, none had a PSD.

About half the CPPs met the inclusion target and another few came close, but the proportion included varied considerably between CPPs. In our retrospective study, we could use individual level register data, readily available in the cancer register. However, there is a delay before these data become available, due to e. g. reporting, data checking and transfer. There is also an official near real-time evaluation of the target^4, 15^. For this to happen, aggregated data are used, and the number of cancer cases are predicted since actual cases of cancer have not yet been reported. Despite methodological differences between our approach and theirs, the results are quite similar (Supplementary figure S2), and any discrepancies are due to inevitable consequences of different methodology and differences between predicted and actual cases of cancer. However, the similarity of these results is encouraging, and although our retrospective approach requires data to be reported and be made available, it enables us to make a more thorough investigation which supplements the official evaluation with additional data and outcomes.

Only five CPPs met the lead time target, though another couple came close. The results vary substantially between CPPs. For many CPPs, the lead times vary by treatment modality, as indicated with ranges in the table. Since we lack data on a patient's actual treatment modality, we make conservative comparisons of lead times against the longest lead time in the range, thus imposing additional uncertainty. In Fig. 3, we show the average investigation time observed in ten CPPs, and hypothetical times had they met the lead time target. The difference (red colour) indicates the excess time due to not meeting the target. The width of each bar indicates the number of patients going through the CPP. If the target was met, the red areas would disappear and this would correspond to a reduced number of patients under investigation each day, and hence a reduced burden for healthcare. Two examples, the Prostate cancer and Lung cancer CPPs have high potential for such reduction. The Kidney cancer CPP has a high excess investigation time, but few patients and the potential reduction is therefore low. As noted above, the Urothelial cancer CPP appears to include patient generously and often rejects the suspicion of cancer (Fig. 2c), but the potential reduction of investigation time is modest. The Breast cancer CPP (Fig. 2b) with filter function and a high share of patients starting cancer treatment has approximately the same potential, despite the different appearances of these two CPPs.

Our illustration does not show the cost of investigation, only the patient volume and a theoretical potential for reducing investigation time. Unless the cost of investigation indicates the opposite, the inclusion criteria of the Urothelial CPP do not strike us as inappropriately generous. Fortunately, this also appears to be the case for the Colorectal cancer CPP. As this is the most common one, any excess resource consumption would have had major negative impact. In this context however, there are several aspects that require our attention. First, excess investigation time increases the patients' waiting times, but does it also correspond to excess use of healthcare resources? It might be the same resource use merely spread over a longer period. On the other hand, the waiting time could be a burdensome time for the patients, fearing a cancer diagnosis but hoping for the opposite. Thus, the excess time could be associated with an avoidable quality of life decrement. Our analysis does not show if the excess time affects a patient's prognosis, just the process leading to cancer dismissal or diagnosis. Still, we can view this partial picture. The inclusion criteria for CPPs are regularly discussed and reviewed if necessary. The wider the inclusion, the greater the chance of discovering cancer but also the more individuals requiring investigation, potentially resulting in a period of worry and lower quality of life. A high proportion of suspicion rejected could correspond to unnecessary quality of life loss^5^. A wider inclusion could discover cancer earlier and improve the prognosis, compared to awaiting symptomatic cancer. Later stages of cancer mean worse prognosis for the patient and often require more intensive treatment. Investigation of more patients requires more resources, and this increases costs, but earlier discovery of cancer might offset treatment costs, and a better prognosis appears undeniably beneficial. A comprehensive evaluation of costs and quality of life associated with the use of CPPs and their inclusion criteria appears a complex but important undertaking. We suggest that in discussions of resource allocation and efficiency of cancer care, the role of CPP inclusion criteria is given attention.

There are to our knowledge only a few studies of costs and effectiveness associated with the implementation of CPPs, and none of their design. Knudsen et al. found that implementation of CPPs i Denmark in 2008 was regarded as having good effect, but observed that supportive evidence was still lacking^17^. They note indications of positive effects, the share of patients starting on treatment within lead time has increased, previously observed low survival in Denmark has caught up with the other Nordic countries and there are allegedly fewer barriers to diagnosis and treatment. Implementation of CPPs in Norway started in 2015, like in Sweden. This increased the demands on healthcare though there were no additional allocation of resources. Careful review of costs and benefits, and proper planning is therefore requested before any new reforms are carried out^18^. Qualitative studies show that patients experience benefits such as predictability and safety associated with CPPs^19^, and comfort from knowing that their CPPs ensures rapid diagnosis and start of treatment^20^. In a study of how implementing a colorectal cancer CPP affected costs, the authors found various effects but increased costs overall, and suggest further follow-up studies of e.g. survival^21^.

We faced a complex analysis, with many CPPs all with their own inclusion criteria and a range of different outcomes combined with numerous different cancer diagnoses. We chose the approach to map the patients into a state-transition model, whose design was a fundamental assumption. We claim however that this approach and its design is reasonable, providing the means to address a complex situation and manage censored data, and visualize the patient event histories. Although CPPs, if used, may be organized quite differently in other countries we would expect the general principles of our visualization nonetheless to be useful. Possibly, it could be applied as is to data from other countries with similar organization of CPPs, and most certainly it could be applied to data from other regions of Sweden. According to official statistics in 2018, the Swedish regions were fairly heterogeneous with regards to the inclusion target, and Region Skåne (78%) was well ahead of the national average (71%)^15^. Data on the lead time target with all CPPs pooled showed less heterogeneity among regions, and Region Skåne (41%) was slightly lower than the national average (45%). Looking at individual CPPs, five CPPs in Skåne were > 10% lower than national average (e. g. urothelial cancer), and four > 10% above. (e. g. Bone and soft tissue sarcoma). We studied cancer cases among patients where a suspicion of cancer was present, and who were investigated through a CPP. We observed cancer cases among those not going through a CPP indirectly when we studied the inclusion target. Among those going through a CPP, we only included those who underwent exactly one CPP during 2018, i. e. we excluded the complex cases going through more than one, in parallel or in sequence. We plan however to study those cases in a follow-up study to generate valuable insights into cases with unspecific symptoms or difficult to investigate for other reasons.

## Conclusions

- Our visualization of the patient flow offers an overview picture of the investigation time and the outcomes of the standardized cancer patient pathways.
- The standardized cancer patient pathway is an effective approach to discover cancer. In cases where suspicion of cancer had been rejected, the primarily suspected diagnosis was very uncommon, as was other cancer diagnoses.
- We did not observe the primarily suspected diagnosis in any of the cases not meeting the criteria for well-founded suspicion.

### Supplementary Information

Supplementary Information.

## Data Availability

The data underlying this study are confidential. For inquiries, please contact the corresponding author.

## Abbreviations

CPP: Standardized cancer patient pathway
CUP: Cancer with unknown primary tumour (CPP)
PSD: Primarily suspected diagnosis
WFS: Well-founded suspicion
