# Supplementary material for: Performance of standardized cancer patient pathways in Sweden visualized using observational data and a state-transition model
Source: Sci Rep. 2023 Nov 9;13:19535. doi: 10.1038/s41598-023-46757-x (PMC10636179; doi:10.1038/s41598-023-46757-x)
Supplement: Supplementary file 1 — Supplementary Information. [file 41598_2023_46757_MOESM1_ESM.docx]

Supplementary material

# Technical appendix: A1. The state-transition model

Our model was implemented as a Markov chain (Ross, 1988), with up to three tunnel states to accommodate transition probabilities dependent on time since entry (Borg et al, 2010), and the model is shown in Figure 1. The escape probability in each state was estimated with Kaplan-Meier to deal with censoring at end of 2018. From this point we did not have data on subsequently started CPPs. The escape probabilities were then used to estimate transition probabilities using a least square method. The model used 14-day cycles.

The states were used to visualize the population movement from start of a CPP to the CPP endpoint, e. g. start of cancer treatment, suspicion of cancer rejected, other cancer, and downstream states such as cancer despite suspicion had previously been rejected, and death. The patient flow describes, in each point in time, the shares of the population visiting each state. We visualized the flow in a six-month period following well-founded suspicion. Due to lack of data the flow does not show further downstream events like end of cancer treatment and relapses.

# References

Borg S, Persson U, Jess T, Thomsen OO, Ljung T, Riis L, et al. A maximum likelihood estimator of a Markov model for disease activity in Crohn's disease and ulcerative colitis for annually aggregated partial observations. Medical Decision Making. 2010;30(1):132-42.

Ross SM. Introduction to probability models. 4th ed. Boston: Academic Press; 1989. xiv, 544 p. p.

**Table S1**: The number of patients going through each standardized cancer clinical pathway (CPP) in 2018, and CPP'S demographic profile.

| **CPP** | **As a single CPP** | **As one of multiple CPPs, n (%)*** | | **Men (%)** | **Age mean (SD)** |
| --- | --- | --- | --- | --- | --- |
| Colorectal cancer | 3 278 | 422 | (11) | 46 | 67.6 (13.2) |
| Urothelial cancer | 2 550 | 214 | (8) | 59 | 68.8 (11.4) |
| Breast cancer | 2 512 | 121 | (5) | 2 | 55.6 (16.5) |
| Prostate cancer | 2 155 | 152 | (7) | 100 | 67.0 (9.0) |
| Melanoma | 1 668 | 156 | (9) | 52 | 62.8 (17.1) |
| Lung cancer | 944 | 233 | (20) | 48 | 70.3 (10.9) |
| Head and neck cancer | 678 | 121 | (15) | 52 | 63.6 (16.4) |
| Uterine cancer | 347 | 41 | (11) | 0 | 65.3 (12.4) |
| Nonspecific symptoms possibly indicative of cancer | 289 | 122 | (30) | 46 | 65.5 (13.4) |
| Pancreatic cancer | 249 | 39 | (14) | 49 | 71.8 (9.7) |
| Kidney cancer | 231 | 73 | (24) | 65 | 67.4 (12.8) |
| Lymphoma | 221 | 129 | (37) | 52 | 61.7 (17.8) |
| Cancer with unknown primary tumour | 220 | 144 | (40) | 38 | 68.3 (13.0) |
| Oesophageal and stomach cancer | 191 | 56 | (23) | 70 | 71.7 (10.6) |
| Brain tumour | 145 | 14 | (9) | 46 | 59.8 (16.5) |
| Bone and soft tissue sarcoma | 124 | 21 | (14) | 54 | 55.9 (15.6) |
| Testicular cancer | 118 | 6 | (5) | 100 | 43.8 (17.2) |
| Thyroid cancer | 114 | 22 | (16) | 22 | 51.4 (16.2) |
| Cervix cancer | 95 | 8 | (8) | 0 | 51.7 (14.6) |
| Liver cancer | 92 | 25 | (21) | 63 | 66.1 (11.8) |
| Myeloma | 88 | 30 | (25) | 53 | 73.5 (9.1) |
| Hepatobiliar cancer | 80 | 20 | (20) | 51 | 69.1 (11.9) |
| Anal cancer | 55 | 23 | (29) | 29 | 65.8 (14.5) |
| Penile cancer | 48 | 4 | (8) | 100 | 68.7 (16.0) |
| Vulvar cancer | 42 | 3 | (7) | 0 | 73.6 (12.0) |
| Acute leukaemia | 34 | 0 | (0) | 59 | 64.9 (15.8) |
| Abdominal and gynaecological sarcoma | 19 | 11 | (37) | 53 | 63.6 (14.2) |
| Neuroendocrine tumours | 9 | 3 | (25) | 89 | 64.9 (12.9) |

Notes: * Proportion among patients going through this particular CPP. Overall in patients going through any CPP, single or multiple, the proportion going through more than one CPP was 6%.

**Figure S1a:** Patient flow in each of the standardized cancer patient pathways (CPP).

Colorectal cancer CPP Urothelial cancer CPP Breast cancer CPP

Prostate cancer CPP Melanoma CPP Lung cancer CPP

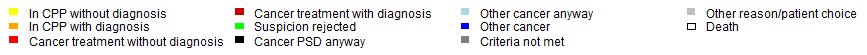


Notes: All 28 CPPs are shown in the figures S1a-S1e. PSD = Primarily Suspected cancer Diagnosis. For defintion of states, see Figure 1.

**Figure S1b:** Patient flow in each of the standardized cancer patient pathways (CPP).

Head and neck cancer CPP Uterine cancer CPP Nonspecific symptoms possibly indicative of cancer CPP

Pancreatic cancer CPP Kidney cancer CPP Lymphoma CPP

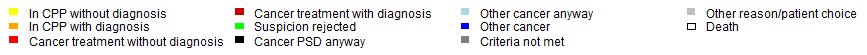


Notes: All 28 CPPs are shown in the figures S1a-S1e. PSD = Primarily Suspected cancer Diagnosis. For defintion of states, see Figure 1.

**Figure S1c:** Patient flow in each of the standardized cancer patient pathways (CPP).

Cancer with unknown primary tumour Oesophageal and stomach cancer CPP Brain tumour CPP

Bone and soft tissue sarcoma CPP Testicular cancer CPP Thyroid cancer CPP

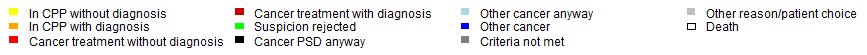


Notes: All 28 CPPs are shown in the figures S1a-S1e. PSD = Primarily Suspected cancer Diagnosis. For defintion of states, see Figure 1.

**Figure S1d:** Patient flow in each of the standardized cancer patient pathways (CPP).

Cervical cancer CPP Liver cancer CPP Myeloma CPP

Hepatobiliar cancer CPP Anal cancer CPP Penile cancer CPP

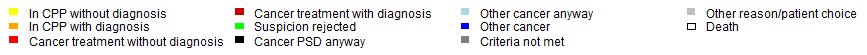


Notes: All 28 CPPs are shown in the figures S1a-S1e. PSD = Primarily Suspected cancer Diagnosis. For defintion of states, see Figure 1.

**Figure S1e:** Patient flow in each of the standardized cancer patient pathways (CPP).

Vulvar cancer CPP Acute leukaemia CPP Abdominal and gynaecological sarcoma

Neuroendocrine tumours CPP

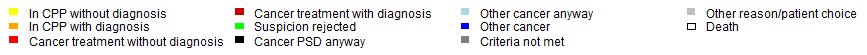


Notes: All 28 CPPs are shown in the figures S1a-S1e. PSD = Primarily Suspected cancer Diagnosis. For defintion of states, see Figure 1.

**Figure S2:** Inclusion target comparison of retrospective analysis (blue) and official near real-time monitoring* (orange).

Notes: * the near real-time monitoring uses predicted number of cancer cases due to delay in reporting to the Swedish cancer registry.

**Figure S3:** Patient flow in standardized cancer patient pathways (CPP), stratified by sex, age, and presence of filter function in the CPP.

(a) Men (c) Patients < 70 years (e) Patients in CPPs without filter function


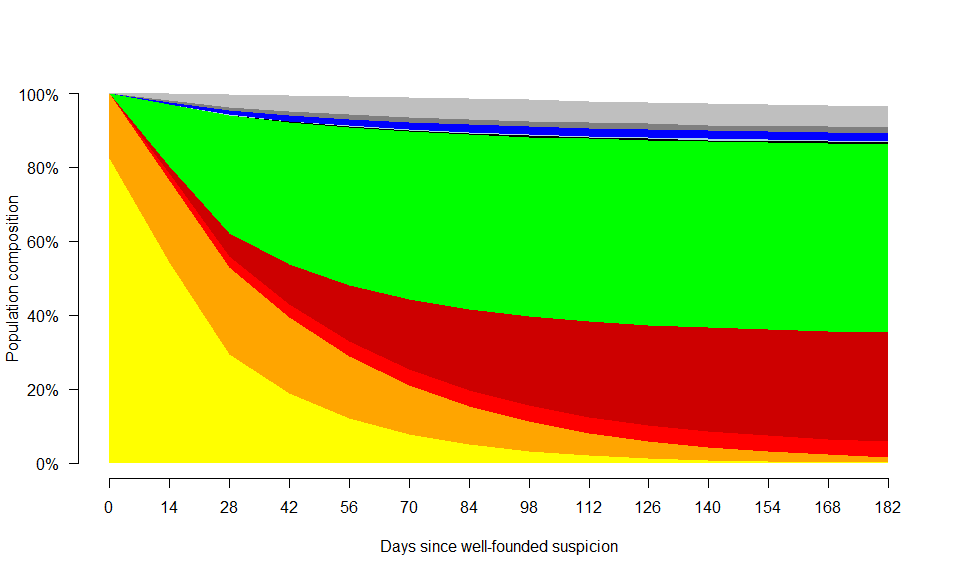

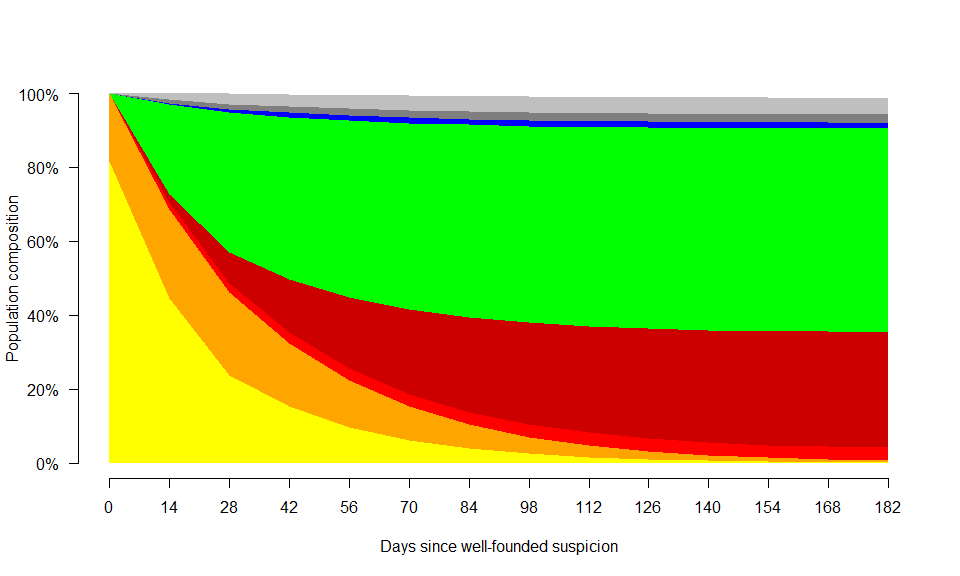

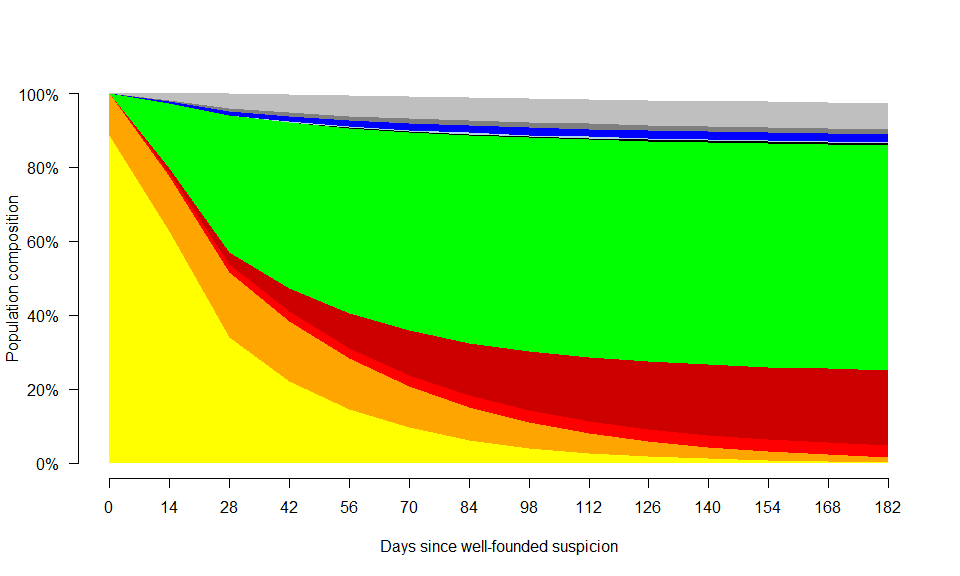


(b) Women (d) Patients ≥ 70 years (f) Patients in CPPs with filter function


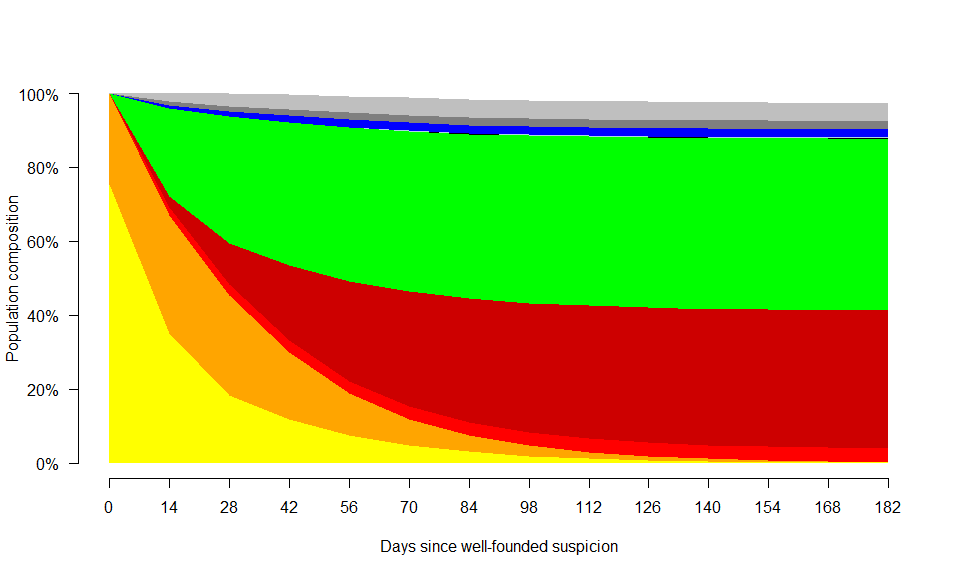

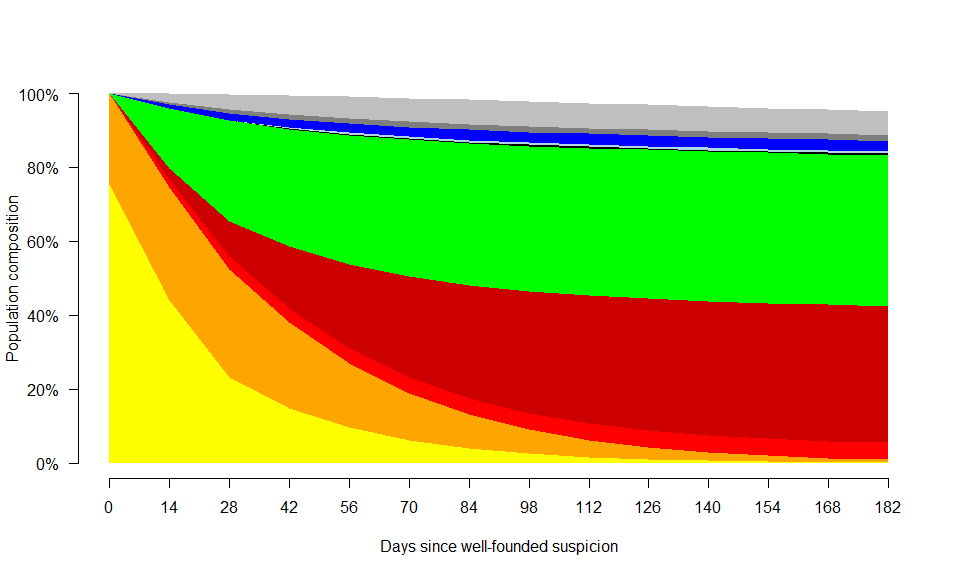

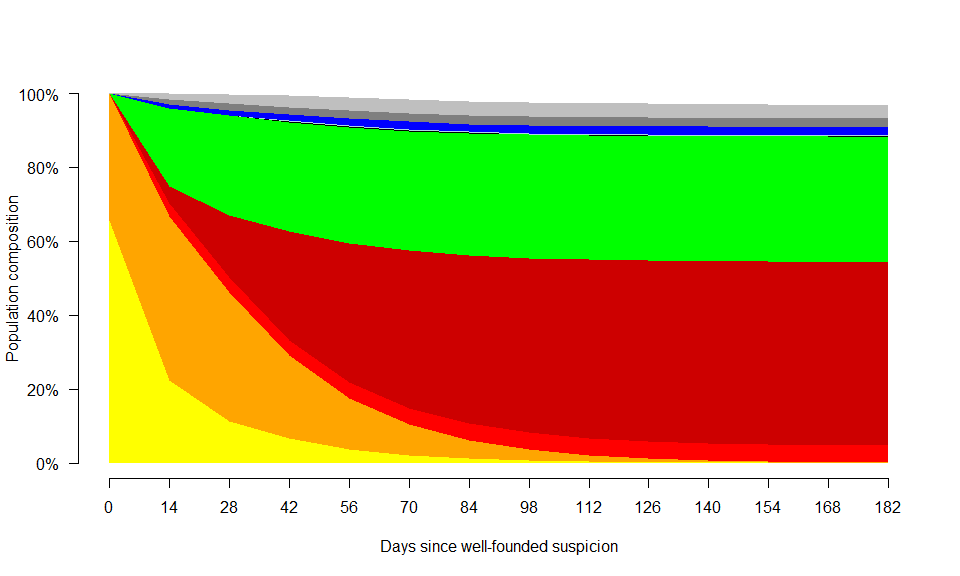


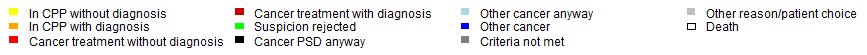


Notes: All 28 CPPs pooled before stratification. PSD = Primarily Suspected cancer Diagnosis. For defintion of states, see Figure 1.

**Table S2**: Number of patients, sex and age in standardized cancer patient pathways (CPPs) and in subgroups by sex, age and by presence of filter function in the CPP.

| **Subgroup** | **n** | **Men (%)** | **Women (%)** | **Age, Mean (SD)** |
| --- | --- | --- | --- | --- |
| Men | 8 154 | 100 | 0 | 67.0 (12.9) |
| Women | 8 442 | 0 | 100 | 62.7 (15.8) |
| < 70 years | 9 391 | 46 | 54 | 55.0 (11.4) |
| ≥ 70 years | 7 205 | 53 | 47 | 77.6 (5.7) |
| In CPPs with filter function | 9 488 | 62 | 38 | 67.4 (12.2) |
| In CPPs without filter function | 7 108 | 32 | 68 | 61.3 (16.6) |
| **All patients** | 16 596 | 49 | 51 | 64.8 (14.6) |

**Table S3**: Observed cancer diagnoses after investigation in each standardized cancer patient pathway (CPP)*.

| CPP | Colorectal cancer | Urothelial cancer | Breast cancer | Prostate cancer | Melanoma | Lung cancer | Head and neck cancer | Uterine cancer | Pancreatic cancer | Kidney cancer | Lymphoma | Oesophageal and stomach cancer | Brain tumour | Bone and soft tissue sarcoma | Testicular cancer | Thyroid cancer | | Cervix cancer | Liver cancer | Myeloma | Hepatobiliar cancer | Anal cancer | Penile cancer | Vulvar cancer | Acute Lymphocytic Leukaemia | Acute Myeloid Leukaemia | Abdominal and gynaecological sarcoma | Neuroendocrine tumours | Other | C80.9 |
| --- | --- | --- | --- | --- | --- | --- | --- | --- | --- | --- | --- | --- | --- | --- | --- | --- | --- | --- | --- | --- | --- | --- | --- | --- | --- | --- | --- | --- | --- | --- |
| Colorectal cancer | **507** | 1 |  | 6 | 1 | 3 |  |  |  | 1 | 2 |  |  |  |  | |  | 1 |  | 1 |  | 2 |  |  |  |  | 1 | 5 | 93 | 1 |
| Urothelial cancer |  | **277** |  | 13 | 1 |  |  |  | 1 | 1 |  |  |  |  |  | |  |  |  |  |  |  | 1 | 1 |  | 1 | 1 |  | 15 | 1 |
| Breast cancer | 1 |  | **998** |  |  |  |  |  |  |  | 1 |  |  |  |  | |  |  |  |  |  |  |  |  |  |  |  | 1 | 12 | 1 |
| Prostate caner | 1 | 3 |  | **524** | 1 | 2 |  |  |  |  |  | 1 |  |  |  | |  |  |  | 1 |  |  |  |  |  |  |  |  | 17 | 1 |
| Melanoma |  |  |  | 2 | **511** |  |  |  |  |  |  |  |  |  |  | |  |  |  |  |  |  |  |  |  |  |  |  | 103 | 2 |
| Lung cancer | 1 |  | 1 | 1 |  | **132** |  |  | 1 | 1 | 1 |  |  |  |  | |  |  | 1 |  |  |  |  |  |  |  | 1 |  | 11 | 5 |
| Head and neck cancer |  |  |  |  | 1 | 2 | **109** |  |  |  | 7 | 1 |  |  |  | | 4 |  |  |  |  |  |  |  |  |  |  |  | 25 | 9 |
| Uterine cancer |  | 1 |  |  |  |  |  | **67** |  |  |  |  |  |  |  | |  |  |  |  |  |  |  |  |  |  | 8 | 1 | 8 |  |
| Nonspecific symptoms possibly indicative of cancer | 1 | 1 |  | 1 |  | 5 |  |  | 2 |  |  | 1 |  |  |  | | 1 |  |  |  | 2 |  |  |  |  |  |  |  | 9 | 4 |
| Pancreatic cancer |  |  |  |  |  | 1 |  |  | **64** |  |  |  |  |  |  | |  |  |  |  | 14 |  |  |  |  |  |  | 3 | 7 | 2 |
| Kidney cancer | 1 | 2 |  | 1 | 1 | 1 |  |  |  | **28** |  |  |  |  |  | |  |  |  |  |  |  |  |  |  |  |  |  |  | 1 |
| Lymphoma |  |  | 1 |  |  | 2 |  | 1 |  |  | **30** |  |  |  |  | |  |  |  |  |  |  |  |  |  |  |  |  | 7 |  |
| Cancer with unknown primary tumour | 2 |  | 3 | 1 |  | 5 |  |  | 1 | 1 | 2 | 1 |  |  |  | |  |  |  | 1 |  |  |  |  |  | 1 |  | 5 | 17 | 15 |
| Oesophageal and stomach cancer |  |  |  | 1 |  |  |  |  | 1 |  | 3 | **110** |  |  |  | |  |  |  |  |  |  |  |  |  |  | 1 |  | 6 |  |
| Brain tumour |  |  |  |  |  | 2 |  |  |  |  |  |  | **55** |  |  | |  |  |  |  |  |  |  |  |  | 1 |  |  | 8 |  |
| Bone and soft tissue sarcoma |  |  |  |  |  | 1 |  |  |  |  |  |  |  | **5** |  | |  |  |  |  |  |  |  |  |  |  | 1 |  |  |  |
| Testicular cancer |  |  |  |  |  |  |  |  |  |  |  |  |  |  | **41** | |  |  |  |  |  |  |  |  |  |  |  |  |  | 1 |
| Thyroid cancer |  |  |  |  |  |  |  |  |  |  |  |  |  |  |  | | **22** |  |  |  |  |  |  |  |  |  |  |  | 2 |  |
| Cervix cancer |  |  |  |  |  |  |  | 1 |  |  |  |  |  |  |  | | 1 | **19** |  |  |  |  |  |  |  |  |  |  | 2 |  |
| Liver cancer | 1 |  |  |  |  |  |  |  |  |  |  |  |  |  |  | |  |  | **18** |  |  |  |  |  |  |  |  |  | 1 | 1 |
| Myeloma |  | 1 |  |  |  |  |  |  |  |  | 2 |  |  |  |  | |  |  |  | **38** |  |  |  |  |  |  |  |  | 1 |  |
| Hepatobiliar cancer |  |  |  |  |  |  |  |  |  |  |  |  |  |  |  | |  |  | 1 |  | **8** |  |  |  |  |  |  |  | 1 |  |
| Anal cancer |  |  |  |  |  |  |  |  |  |  |  |  |  |  |  | |  |  |  |  |  | **8** |  |  |  |  |  |  | 1 |  |
| Penile cancer |  |  |  |  |  |  |  |  |  |  |  |  |  |  |  | |  |  |  |  |  |  | **9** |  |  |  |  |  |  |  |
| Vulvar cancer |  |  |  |  |  |  |  |  |  |  |  |  |  |  |  | |  |  |  |  |  |  |  | **5** |  |  |  |  | 1 |  |
| Acute leukaemia |  |  |  |  |  |  |  |  |  |  |  |  |  |  |  | |  |  |  |  |  |  |  |  | **3** | **20** |  |  | 2 |  |
| Abdominal and gynaecological sarcoma |  |  |  |  |  |  |  |  |  |  |  |  |  |  |  | |  |  |  |  |  |  |  |  |  |  | **5** |  | 2 |  |
| Neuroendocrine tumours |  |  |  |  |  |  |  |  |  |  |  |  |  |  |  | |  |  |  |  |  |  |  |  |  |  |  | **2** |  | 1 |

Notes: * Diagnoses occurring after suspicion of cancer had been rejected and diagnoses in patients failing to meet CPP inclusion criteria, have been excluded. C80.9 = Malignant neoplasm, primary site unspecified. Boldface indicates the primarily suspected cancer diagnosis in relevant CPPs.
